# Monocyte Distribution Width and Composite Biomarker Assessment for Prognostic Stratification of Sepsis in the Intensive Care Unit

**DOI:** 10.3390/biomedicines14040787

**Published:** 2026-03-30

**Authors:** Jana Arsenijević, Marijana Stanojević Pirković, Dragan R. Milovanovic, Marina Kostić, Biljana Popovska Jovičić, Ivana Lešnjak, Mirela Jevtić, Sara Mijailović, Sanja Knežević, Dušan Radojević, Maja Pešić, Bojan Stojanović, Dragče Radovanović, Olgica Mihaljević, Danijela Jovanović

**Affiliations:** 1 Faculty of Medical Sciences, University of Kragujevac, Svetozara Markovića 69, 34000 Kragujevac, Serbia; 2 Department of Medical Biochemistry, Faculty of Medical Sciences, University of Kragujevac, Svetozara Markovića 69, 34000 Kragujevac, Serbia; 3 Center for Laboratory Diagnostics, University Clinical Center Kragujevac, Zmaj Jovina 30, 34000 Kragujevac, Serbia; 4 Department of Pharmacology and Toxicology, Faculty of Medical Sciences, University of Kragujevac, Svetozara Markovića 69, 34000 Kragujevac, Serbiamarrina2006kg@yahoo.com (M.K.); 5 Clinical Pharmacology Department, University Clinical Centre Kragujevac, Zmaj Jovina 30, 34000 Kragujevac, Serbia; 6 Department of Infectious Diseases, Faculty of Medical Sciences, University of Kragujevac, Svetozara Markovica 69, 34000 Kragujevac, Serbia; 7 Clinic for Infectious Diseases, University Clinical Centre Kragujevac, Zmaj Jovina 30, 34000 Kragujevac, Serbia; 8 Department of Gynaecology and Obstetrics, General Hospital Uzice, Miloša Obrenovića 17, 31000 Uzice, Serbia; 9 Department of Medical Statistics and Informatics, Faculty of Medical Sciences, University of Kragujevac, Svetozara Markovića 69, 34000 Kragujevac, Serbia; 10 Department of Pediatrics, Faculty of Medical Sciences, University of Kragujevac, Svetozara Markovića 69, 34000 Kragujevac, Serbia; 11 Clinic for Pediatrics, University Clinical Center Kragujevac, Svetozara Markovića 69, 34000 Kragujevac, Serbia; 12 Department of Internal Medicine, Faculty of Medical Sciences University of Kragujevac, Svetozara Markovica 69, 34000 Kragujevac, Serbia; 13 Clinic for Gastroenterology and Hepatology, University Clinical Center Kragujevac, Zmaj Jovina 30, 34000 Kragujevac, Serbia; 14 Department of Surgery, Faculty of Medical Sciences, University of Kragujevac, Svetozara Markovića 69, 34000 Kragujevac, Serbia; 15 Clinic for Surgery, University Clinical Center Kragujevac, Zmaj Jovina 30, 34000 Kragujevac, Serbia; 16 Department of Pathophysiology, Faculty of Medical Sciences, University of Kragujevac, Svetozara Markovića 69, 34000 Kragujevac, Serbia; vrndic07@yahoo.com; 17 Department of Anesthesiology and Reanimation, University Clinical Center Kragujevac, Zmaj Jovina 30, 34000 Kragujevac, Serbia

**Keywords:** sepsis, monocyte distribution width, biomarkers, Sequential Organ Failure Assessment, intensive care unit, mortality

## Abstract

**Background**: Sepsis is a life-threatening organ dysfunction caused by a dysregulated host response to infection and remains a leading cause of mortality in intensive care units (ICUs). Although the Sequential Organ Failure Assessment (SOFA) score is widely used for prognostic stratification, organ dysfunction represents a downstream manifestation of sepsis, whereas immune and inflammatory dysregulation may precede overt organ failure. Monocyte distribution width (MDW) is a novel hematological parameter reflecting monocyte activation and is approved for the diagnosis of sepsis; however, its prognostic value and potential role within composite biomarker models in critically ill surgical patients with sepsis remain incompletely defined. **Methods**: We conducted a prospective, observational, single-center pilot study in two surgical intensive care units between November 2022 and December 2023. Adult patients with sepsis defined according to Sepsis-3 criteria were enrolled. Laboratory and clinical variables—including MDW, neutrophil-to-lymphocyte ratio (NLR), C-reactive protein (CRP), procalcitonin (PCT), and SOFA score—were measured on admission and during the first five days of ICU stay. Patient-level median values across five days were used for analysis. The primary outcome was in-hospital mortality. Prognostic performance was assessed using receiver operating characteristic (ROC) curve analysis and logistic regression. A composite bioscore was constructed by combining dichotomized MDW, NLR, CRP, and PCT values. **Results**: Sixty patients were included; 24 (40%) died during hospitalization. Non-survivors were older and had significantly higher SOFA scores. MDW, NLR, CRP, and PCT were significantly higher in non-survivors. SOFA demonstrated the strongest discriminative ability for mortality prediction (AUC 0.839, 95% CI 0.730–0.948). Among biomarkers, NLR (AUC 0.741) and PCT (AUC 0.714) showed good discriminative performance, while MDW (AUC 0.690) and CRP (AUC 0.662) showed moderate discrimination; MDW exhibited the highest specificity (80.6%). In multivariable analysis with individual biomarkers, only SOFA remained an independent predictor of mortality. The composite bioscore demonstrated good discriminative ability (AUC 0.805) and, when evaluated alongside SOFA, remained independently associated with fatal outcome (OR 11.92, 95% CI 1.76–80.75); however, given the modest sample size and wide confidence intervals, this finding should be interpreted with caution. Repeated-measures correlation analysis revealed no strong collinearity among biomarkers. **Conclusions**: A composite bioscore incorporating MDW, NLR, CRP, and PCT provides prognostic information comparable to SOFA and remains independently associated with mortality. This approach may complement organ dysfunction-based assessment and support early risk stratification in sepsis.

## 1. Introduction

Sepsis is a life-threatening organ dysfunction caused by a dysregulated host response to infection, as defined by the Sepsis-3 consensus, and remains a leading cause of morbidity and mortality worldwide, accounting for approximately 49 million cases and 11 million deaths annually [1,2]. Timely recognition and appropriate management of sepsis are critical determinants of patient outcomes, making prognostic stratification during ICU admission a central component of intensive care practice [3]. However, despite advances in therapy, sepsis is characterized by marked biological heterogeneity, and the trajectory of clinical deterioration may not be adequately reflected by isolated measurements at a single time point, limiting the effectiveness of organ failure-based assessment when applied statically [4]. Consequently, there is an ongoing need for reliable biomarkers that can support prognostic assessment across the early course of ICU stay in critically ill patients.

An ideal sepsis biomarker should be readily available, sensitive to immune and inflammatory dysregulation, and capable of providing prognostic information beyond established clinical severity scores—ideally when measured serially over the initial days of ICU admission. Among currently used biomarkers, PCT is widely applied for diagnostic and prognostic purposes in sepsis. However, its limited sensitivity in certain clinical settings may reduce its reliability as a standalone marker [5]. These limitations have stimulated interest in alternative or complementary biomarkers derived from routine laboratory testing. Increasing evidence suggests that parameters obtained from the complete blood count reflect key aspects of the host immune response during infection and sepsis [6]. One such parameter is MDW, which reflects changes in circulating monocyte volume associated with activation of pro-inflammatory pathways in response to pathogen-associated stimuli. Previous studies have demonstrated that MDW shows high sensitivity and negative predictive value for sepsis, particularly in emergency department populations, whereas PCT tends to exhibit higher specificity [7]. However, data regarding the prognostic role of MDW in critically ill ICU populations, particularly when assessed longitudinally, remain limited.

Beyond individual biomarkers, there is growing recognition that combined biomarker approaches, particularly when integrated with clinical severity scores, may improve prognostic assessment in sepsis. Composite models incorporating inflammatory and hematological indices—such as NLR, platelet-to-lymphocyte ratio (PLR), CRP, PCT, and the SOFA score—have demonstrated improved performance compared with single markers in various clinical settings [8,9,10]. These findings support the concept that sepsis severity and outcome are unlikely to be adequately captured by a single biological parameter. Despite these advances, it remains unclear which combinations of routinely available biomarkers provide the most clinically useful balance between prognostic accuracy and practical applicability in the ICU. In particular, the added value of MDW when combined with other inflammatory markers and evaluated alongside SOFA over the initial days of ICU stay has not been sufficiently explored in surgical ICU populations.

Therefore, the aim of this pilot study was to explore whether a composite bioscore incorporating routinely available inflammatory and hematological biomarkers including PCT, CRP, and NLR among others, can match the prognostic performance of SOFA and outperform individual markers for predicting in-hospital mortality in surgical ICU patients with sepsis, based on serial measurements obtained during the first five days of ICU admission. The bioscore is intended as a longitudinal prognostic tool reflecting the trajectory of host immune and inflammatory response across the initial phase of ICU stay, rather than a single-timepoint admission decision aid. As such, the present findings are hypothesis generating, with external validation in larger, multicenter cohorts required before clinical implementation can be recommended.

## 2. Materials and Methods

### 2.1. Study Design and Population

This prospective, observational, exploratory, non-interventional, single-center pilot study was conducted in two surgical intensive care units (ICUs) at the University Clinical Center Kragujevac between November 2022 and December 2023. Adult patients (≥18 years) with sepsis diagnosed according to Sepsis-3 criteria (SOFA score ≥ 2) were eligible for inclusion. Exclusion criteria were age < 18 years, pregnancy, immunocompromised status (including liver failure, autoimmune diseases, hematological malignancies, organ transplantation, or AIDS), terminal or severe chronic illness (advanced or metastatic cancer, chemotherapy, advanced congestive heart failure, or stroke). The study was conducted in accordance with the Declaration of Helsinki and approved by the Ethics Committee of the University Clinical Center Kragujevac (approval no. 01/22-386). Demographic data, medical history, and clinical findings were collected by trained medical staff. A comprehensive clinical examination was performed at ICU admission (baseline—Day 1) and repeated daily over the subsequent four days (Days 2–5).

### 2.2. Blood Sample Collection and Laboratory Analysis

Venous blood samples were collected at ICU admission and daily for the subsequent four days for routine laboratory analyses. Serial laboratory and clinical assessments—including complete blood count with differential, MDW, CRP, PCT and the Sequential Organ Failure Assessment (SOFA) score—were obtained at ICU admission and repeated daily over the same period. For statistical analyses, patient-level median values across the first five ICU days were calculated and used.

All laboratory analyses were performed at the Center for Laboratory Diagnostics, University Clinical Center Kragujevac, using standard methods in accordance with Good Laboratory Practice. Blood samples for complete blood count and MDW determination were collected in K_2_EDTA tubes. Blood samples for CRP and PCT measurements were collected in serum separator tubes and analyzed using standard biochemical and immunoassay methods. White blood cell counts were measured using the DxH 900 Hematology Analyzer (Beckman Coulter, Brea, CA, USA), biochemical parameters using the DxC 700 Analyzer (Beckman Coulter, USA), and PCT concentrations using the Cobas e 411 immunoassay system (Roche Diagnostics, Mannheim, Germany). Quality assurance procedures included regular internal and external quality controls. Reference ranges were as follows: leukocytes 3.70–10.0 × 10^9^/L, neutrophils 2.10–6.50 × 10^9^/L, lymphocytes 1.20–3.40 × 10^9^/L, platelets 135–450 × 10^9^/L, CRP < 5.0 mg/L, and PCT < 0.5 ng/mL.

### 2.3. Outcomes and Variables

The primary outcome was in-hospital mortality, defined as death during hospital stay and analyzed as a binary variable. The primary independent variable was monocyte distribution width (MDW), analyzed as a continuous variable. Other variables were considered secondary or potential confounders, including age, sex, the SOFA score, and inflammatory biomarkers.

### 2.4. Sample Size Calculation

Sample size calculation was based on the assumption that the expected difference in MDW between outcome groups would be 6 units, with a standard deviation of 7, derived from previously published data [7]. With a power of 0.80, alpha error of 0.05, and an assumed group ratio of 1:3, the minimum required sample size was 45 patients [11].

### 2.5. Statistical Analysis

Statistical analysis included descriptive statistics, with repeatedly measured continuous variables over the first five ICU days summarized as patient-level medians (IQR) to reduce short-term fluctuations and intra-individual variability. Between-group differences were assessed using the independent samples *t*-test or Mann–Whitney U test, as appropriate, and categorical variables using the χ^2^ test. Discriminative performance for mortality was evaluated using ROC analysis, reporting AUC with 95% CI and deriving cut-offs for exploratory purposes.

A composite bioscore was constructed by dichotomizing MDW, NLR, CRP, and procalcitonin using ROC-derived cut-offs and summing points. Associations with mortality were assessed using univariable and multivariable logistic regression. To evaluate robustness and potential information loss due to dichotomization, multivariable analysis was repeated using continuous biomarker values, and internal validation was performed using bootstrapping with 1000 resamples. Incremental prognostic value beyond the SOFA score was assessed using net reclassification improvement (NRI) and integrated discrimination improvement (IDI). Collinearity among biomarkers was assessed using repeated-measures correlation (rmcorr). All tests were two-tailed, and a *p*-value < 0.05 was considered statistically significant. The statistical analysis for this study was conducted using IBM SPSS v22.

## 3. Results

### 3.1. Study Population

Of the 71 patients screened, 11 were excluded owing to incomplete data, leaving a final cohort of 60 patients. Finally, our study included 60 patients with sepsis admitted to the ICU, of whom 36 survived and 24 died during hospital stay. Patients were classified as survivors or non-survivors according to in-hospital outcome. All patients underwent serial laboratory and clinical assessment, including complete blood count with differential (including MDW), CRP, PCT and parameters required for SOFA score calculation, obtained on ICU admission and during four consecutive days. All patients met Sepsis-3 criteria and had bacterial sepsis (ICD-10 codes A40, A41, T81). Sepsis originated from abdominal, pulmonary, skin and soft tissue, urogenital, and musculoskeletal sources, predominantly related to surgical pathology. Baseline demographic and clinical characteristics are summarized in Supplementary Table S1. For analysis, patient-level median values across the first five days of ICU stay were used.

Patients who died were older than survivors (median 72.5 vs. 67 years, *p* = 0.039) and had significantly higher SOFA scores (median 10.2 vs. 4.8, *p* < 0.001). Non-survivors also demonstrated significantly higher levels of MDW, PCT, CRP, NLR and creatinine and a lower lymphocyte count. (Supplementary Table S1).

### 3.2. ROC Curve Analyses

ROC curve analysis was performed for exploratory assessment of discriminative performance for variables that differed significantly between survivors and non-survivors (Table 1). The SOFA score showed the strongest discriminative ability for mortality prediction (AUC 0.839, 95% CI 0.730–0.948), with balanced sensitivity (78.9%) and specificity (75.8%). Among biomarkers, NLR and PCT demonstrated good discriminative performance, with AUCs of 0.741 (95% CI 0.596–0.887) and 0.714 (95% CI 0.584–0.844), respectively. MDW and CRP showed moderate discriminative ability, with AUCs of 0.690 (95% CI 0.543–0.837) and 0.662 (95% CI 0.519–0.805). PCT exhibited the highest sensitivity (91.7%), whereas MDW demonstrated the highest specificity (80.6%) at the optimal cut-off (Table 1).

### 3.3. Logistic Regression Analyses

Univariable logistic regression confirmed that SOFA, MDW, NLR, PCT and CRP were significantly associated with mortality, while PLR was not (Table 2). In the multivariable model, only the SOFA score remained independently associated with fatal outcome in the primary multivariable model (OR 13.54, 95% CI 1.56–117.39; *p* = 0.018). The model demonstrated good calibration (Hosmer–Lemeshow *p* = 0.581) and substantial explained variance (Nagelkerke R^2^ = 0.675) (Table 2). Internal validation using bootstrapping with 1000 resamples demonstrated wide confidence intervals for several biomarkers, indicating limited stability of regression estimates. When multivariable analysis was repeated using continuous biomarker values, only the SOFA score and neutrophil-to-lymphocyte ratio (NLR) remained significantly associated with mortality, while other biomarkers were not independently associated.

### 3.4. Composite Bioscore Analysis

**Figure 1:**
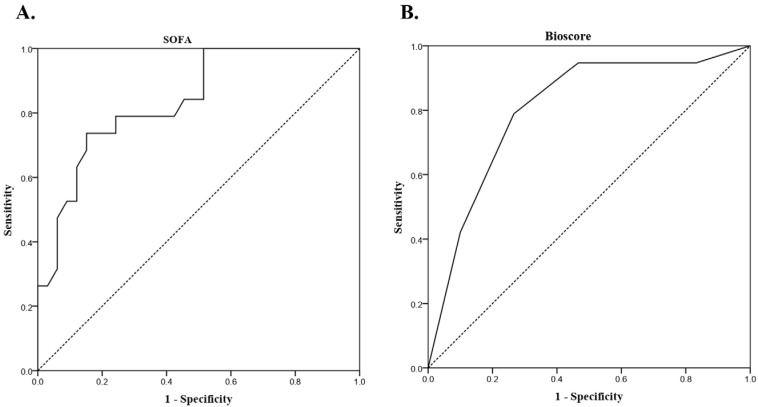
ROC curves for predicting in-hospital mortality: (**A**) SOFA score (AUC 0.839, 95% CI 0.730–0.948; optimal cut-off ≥ 6.5) and (**B**) composite bioscore (AUC 0.805, 95% CI 0.677–0.934; optimal cut-off ≥ 3).

To evaluate whether a combination of biomarkers could improve prognostic performance independently of SOFA, MDW, NLR, CRP, and procalcitonin were dichotomized according to ROC-derived cut-offs and combined into a composite bioscore ranging from 0 to 4 points. The composite bioscore demonstrated good discriminative ability for mortality prediction (AUC 0.805, 95% CI 0.677–0.934; *p* < 0.001) (Figure 1). An optimal cut-off of ≥3 points yielded a sensitivity of 78.9% and specificity of 73.3%. Patients with a composite bioscore ≥ 3 had a significantly higher risk of fatal outcome. In a multivariable model including both SOFA (≥6.5) and the composite bioscore (≥3), both parameters remained associated with mortality in the multivariable model. SOFA ≥ 6.5 was associated with a 14-fold increase in odds of death (OR 14.63, 95% CI 2.20–97.25; *p* = 0.006), while a composite bioscore ≥3 conferred an approximately 12-fold increased risk (OR 11.92, 95% CI 1.76–80.75; *p* = 0.011) (Table 3). Reclassification analysis demonstrated that the addition of the composite bioscore to the SOFA score improved overall model discrimination. Continuous NRI was 1.16 (95% CI 0.68–1.64; *p* < 0.001), and IDI was 0.20 (95% CI 0.10–0.30; *p* < 0.001). However, categorical NRI was not statistically significant (NRI = −0.04; 95% CI −0.33 to 0.26; *p* = 0.81), indicating no significant improvement in classification across predefined risk categories. Comparison of ROC curves using DeLong’s test showed that the model combining SOFA and the composite bioscore had significantly better discrimination than SOFA alone (AUC 0.89 vs. 0.81; Z = −2.25, *p* = 0.025).

### 3.5. Collinearity Assessment

Repeated-measures correlation analysis revealed only weak-to-moderate correlations among biomarkers included in the composite bioscore, with no correlation coefficient exceeding |r| = 0.5, supporting their concurrent inclusion in multivariable and composite models (Supplementary Table S2).

## 4. Discussion

In this study, we evaluated the prognostic performance of monocyte distribution width (MDW), classical inflammatory biomarkers (CRP and PCT), hematological ratios (NLR and PLR), and the SOFA score for predicting mortality in patients with sepsis. MDW, NLR, CRP and PCT were significantly associated with mortality in univariable logistic regression analyses and demonstrated moderate-to-good discriminative ability in ROC curve analyses. None of these biomarkers retained independent prognostic significance after adjustment for SOFA in the primary model; however, in analyses using continuous biomarker values, NLR remained independently associated with mortality. This finding highlights the limitations of relying on single biological markers to capture the complex and heterogeneous pathophysiology of sepsis. Accordingly, we constructed a composite bioscore incorporating MDW, NLR, CRP and PCT, which demonstrated prognostic performance for in-hospital mortality comparable to that of the SOFA score in patients with sepsis.

Among the evaluated biomarkers, PCT and NLR showed good discriminative performance (AUC > 0.70), while MDW and CRP demonstrated moderate discrimination (AUC > 0.65). These markers differed in their sensitivity and specificity profiles, with MDW exhibiting the highest specificity, suggesting potential value in identifying patients at particularly high risk of fatal outcome. Although MDW is well established as a diagnostic marker for sepsis, its prognostic utility remains less clearly defined. In our study, a median MDW value of 29.95 over the first five ICU days discriminated between survivors and non-survivors with moderate sensitivity and high specificity. Previous studies have reported associations between elevated MDW and adverse outcomes using different cut-offs and time points, and have emphasized the prognostic relevance of MDW kinetics during early hospitalization [12,13,14,15]. In our cohort, there was a decrease in MDW in survival group, as opposed to absence of dynamic changes in non-survivor group, but all these changes were not statistically significant. In many previously cited studies, MDW and other inflammatory biomarkers were evaluated without simultaneous adjustment for established clinical severity scores such as SOFA. Conversely, other studies have reported attenuated prognostic performance of MDW after adjustment for clinical severity, likely reflecting heterogeneity in patient populations, analytical approaches, and non-infectious factors known to influence MDW levels, including multimorbidity, metabolic disorders, and differences in anticoagulant use (K_2_EDTA vs K_3_EDTA) [16,17,18,19].

Similarly, NLR demonstrated good prognostic performance in univariable analysis and remained independently associated with mortality in analyses using continuous values, although its effect was attenuated after adjustment for SOFA in the primary model. This suggests that while NLR reflects immune dysregulation in sepsis, part of its prognostic signal overlaps with clinical severity captured by organ dysfunction scores. Previous studies and meta-analyses largely support an association between elevated NLR and mortality and severity of sepsis, although inconsistent findings have also been reported, likely due to differences in study design and timing of measurement [20,21,22,23,24]. CRP and PCT demonstrated significant associations with mortality in univariable analyses, with PCT showing good discriminative ability and the highest sensitivity, while CRP exhibited moderate discriminative performance. In line with previous studies, neither marker retained independent prognostic significance after adjustment for SOFA [24,25,26,27]. This supports the concept that although CRP and PCT reflect systemic inflammation and infection burden, they do not appear to add prognostic information beyond that captured by organ dysfunction-based severity assessment.

The SOFA score showed strong prognostic performance in our cohort, with the median SOFA score over the first five ICU days demonstrating good discrimination for mortality. These findings are consistent with prior studies validating SOFA as a robust prognostic tool [28,29,30,31,32,33]. Moreover, dynamic changes in SOFA were particularly informative in our cohort, as patients with fatal outcomes exhibited increasing scores over time, whereas SOFA remained stable among survivors. Previous studies have emphasized that dynamic SOFA assessment provides greater prognostic value than a single baseline measurement. De Grooth et al. demonstrated that longitudinal SOFA trajectories outperform static scores, while Karakike et al. reported that a failure to achieve a ≥25% reduction in SOFA by day 7 was associated with a markedly increased risk of mortality (OR = 14.87) [30,31]. In a large ICU cohort of more than 20,000 patients, Soo et al. further showed an approximately linear increase in hospital mortality with rising SOFA scores, reinforcing the strong relationship between organ dysfunction burden and outcome [34]. The dominance of the SOFA score in multivariable analysis was expected, as organ dysfunction represents the final common pathway of sepsis-related immune activation, dysregulation, and systemic inflammation. Given the complexity and heterogeneity of sepsis, it is therefore unlikely that disease severity or prognosis can be adequately captured by a single biomarker alone.

Recent research has increasingly focused on the development of multimarker scoring systems (composite bioscores) that integrate biological markers, with or without clinical variables, to improve prognostic stratification and clinical decision-making in sepsis. Composite bioscores incorporating inflammatory and infection-related biomarkers such as presepsin, CRP, lactate, and related parameters have demonstrated superior mortality prediction compared with individual markers in adult ICU populations. ROC curve and logistic regression analyses were therefore used to identify biomarkers with prognostic relevance and to construct a composite bioscore intended to complement organ dysfunction– based risk stratification [9]. In order to construct a composite bioscore, we included parameters that individually demonstrated moderate predictive ability based on ROC curve analysis and univariate logistic regression, with the aim of improving prediction of sepsis outcomes. The composite bioscore included MDW, NLR, PCT and CRP. From an immunological perspective, this combination represents markers reflecting cellular activation (MDW), immune dysfunction (NLR), and systemic inflammatory and infection-related responses (CRP and PCT). From a statistical perspective, this combination is notable for integrating parameters with high specificity (MDW) and high sensitivity (NLR, CRP, and PCT). The composite bioscore demonstrated good discriminative ability for mortality prediction, comparable to that of the SOFA score. In the primary multivariable model, the bioscore remained associated with mortality alongside SOFA; however, internal validation indicated limited stability of regression estimates, and these findings should be interpreted with caution. Reclassification analysis showed that the addition of the bioscore improved continuous measures of risk discrimination, but did not significantly improve classification across predefined risk categories, suggesting limited impact on clinically relevant decision thresholds. While the bioscore integrates complementary aspects of the host response, including cellular activation, immune dysregulation, and systemic inflammation, its incremental prognostic value beyond established clinical scores appears modest and requires validation in larger cohorts. Capturing early biological alterations in sepsis through four routinely measured biomarkers, prior to the onset of overt organ failure as defined by the SOFA score, and leveraging their widespread clinical availability, supports the potential applicability of this composite bioscore in clinical settings.

This study has several limitations, including its single-center design and relatively limited sample size, which may affect generalizability and the stability of multivariable estimates, as reflected by wide confidence intervals. In addition, the number of events relative to the number of predictors may have affected the robustness of regression analyses. External validation was not performed, and the findings should therefore be considered exploratory. Additionally, the exclusion of immunocompromised patients may have selected a less complex patient population, and whether the bioscore performs similarly in these higher-risk groups remains to be established. Nevertheless, the heterogeneity of sepsis sources and patient comorbidities reflects real-world ICU populations and may enhance the applicability of the composite bioscore across different clinical scenarios. Although a weighted bioscore could offer improved statistical performance, the simplicity and interpretability of the present bioscore represent potential advantages, but require confirmation in larger, independent cohorts before clinical implementation.

## 5. Conclusions

Our study demonstrated that a composite bioscore incorporating MDW, NLR, CRP, and PCT achieved prognostic performance for fatal outcome prediction comparable to that of the SOFA score during the first five days in the ICU. The relatively small and heterogeneous study population precluded the development of a more complex, weighted bioscore. Future large-scale, multicenter, controlled studies are warranted to develop continuous or weighted bioscores that may improve effect estimates, as well as sensitivity and specificity for mortality prediction. These findings should nonetheless be interpreted with caution, as the limited sample size and correspondingly wide confidence intervals preclude definitive conclusions regarding the prognostic equivalence of the bioscore and the SOFA score.

## Figures and Tables

**Table 1:** ROC curve analysis of clinical and laboratory parameters for in-hospital mortality prediction.

Parameter	Cut Off	AUC (95% CI)	Sensitivity	Specificity	*p*
SOFA	6.5	0.839 (0.730–0.948)	0.789	0.758	<0.001
MDW	29.95	0.690 (0.543–0.837)	0.625	0.806	0.013
NLR	9.25	0.741 (0.596–0.887)	0.842	0.600	0.005
PLR	254.0	0.611 (0.441–0.782)	0.632	0.500	0.193
CRP	70	0.662 (0.519–0.805)	0.833	0.457	0.036
PCT	1.45	0.714 (0.584–0.844)	0.917	0.556	0.005

**Table 2:** Univariable and multivariable logistic regression analyses for in-hospital mortality.

	Univariate Logistic Regression		Multivariate Logistic Regression	
Parameter	OR (95% CI)	*p*	OR (95% CI)	*p*
SOFA	11.719 (3.007–45.670)	<0.001	13.542 (1.562–117.392)	0.018
MDW	6.905 (2.147–22.202)	0.001	3.690 (0.339–40.143)	0.284
NLR	8.000 (1.908–33.537)	0.004	11.051 (0.845–144.579)	0.067
PLR	1.714 (0.592–5.552)	0.369	-	-
CRP	4.211 (1.191–14.886)	0.026	1.367 (0.085–22.044)	0.825
PCT	8.800 (1.795–43.145)	0.007	6.960 (0.374–129.584)	0.193

**Table 3:** Univariable and multivariable logistic regression analyses including SOFA score and the composite bioscore.

	Univariate Logistic Regression		Multivariate Logistic Regression	
Parameter	OR (95% CI)	*p*	OR (95% CI)	*p*
SOFA ≥ 6.5	11.719 (3.007–45.670)	<0.001	14.628 (2.200–97.248)	0.006
Bioscore ≥ 3	10.312 (2.626–40.500)	0.001	11.915 (1.758–80.752)	0.011

## Data Availability

The original contributions presented in this study are included in the article/Supplementary Materials. Further inquiries can be directed to the corresponding authors.

## Supplementary Materials

The following supporting information can be downloaded at: https://www.mdpi.com/article/10.3390/biomedicines14040787/s1, Table S1. Clinical and laboratory variables of study patients (most significant parameters selected). Table S2. Repeated-measures correlations for combined hematological and biochemical variables in study patients.
