# Supplementary material for: Monocyte Distribution Width and Composite Biomarker Assessment for Prognostic Stratification of Sepsis in the Intensive Care Unit
Source: Biomedicines. 2026 Mar 30;14(4):787. doi: 10.3390/biomedicines14040787 (PMC13113765; doi:10.3390/biomedicines14040787)
Supplement: Supplementary file 1 [file biomedicines-14-00787-s001.zip › biomedicines-4186491-supplementary.pdf]

Supplementary Table S1. Clinical and laboratory variables of study patients (most significant parameters selected)

Variable	All patients (n=60)	Survival group (Controls) (n=36)	Fatality group (Cases) (n=24)	Statistics
Age (years)	69 (19-84, 15, n=60)	67 (19-82, 40, n=36)*	72.5 (47-84, 9, n=24)	U=295.0, p=0.039
Gender (male)	31 (51.7%)	22 (61.1%)	9 (37.5%)	χ^2=3.2, p=0.073
female	29 (48,3%)	14 (38,9%)	15 (62,5%)	
White blood cells (10^9/L)	8.8 (0.5-28.9, 6.2, n=60)	9.2 (2.8-28.9, 5.5, n=36)*	8.2 (0.5-24.4, 8.9, n=24)	U=401.0, p=0.640
Neutrophils (10^9/L)	9.6 (1.9-34.6, 5.7, n=60)	9.6 (3.1-34.6, 5.4, n=36)*	9.6 (1.9-24.2, 9, n=24)*	U=360.0, p=0.277
Lymphocytes (10^9/L)	0.8 (0.2-2.4, 0.6, n=60)	1.0 (0.2-2.3, 0.6, n=36)	0.7 (0.2-2.4, 0.4, n=24)*	U=266.0, p=0.012
NLR (Neutrophils/Lymphocytes)	11.8 (1.7-106.4, 9.7, n=60)*	9.2 (3.1-27.1, 7.0, n=36)*	14.4 (1.7-106.4, 13.1, n=24)*	U=241.0, p=0.004
Platelets (10^9/L)	208.5 (48.4-832.2, 134.6, n=60)*	221 (67.3-832.2, 145.8, n=36)*	194.2 (48.4, 475.3, 133.6, n=24)	U=335.5, p=0.145
PLR (Platelets/Lymphocytes)	277.1 (48.2-749.1, 222, n=60)*	248.1 (48.2-621.8, 155.9, n=36)*	325.8 (89.3-749.1, 312.5, n=24)	U=335.5, p=0.145
MDW (Monocyte distribution width)	28 (19.6-46.3, 8, n=60)*	27.4 (19.6-46.3, 6, n=36)	31.3 (21.0-43.5, n=24)	U=268.0, p=0.013
CRP (C-reactive protein (mg/L))	104.6 (1.6-390.9, 114.1, n=60)*	87.2 (1.6-390.9, 111.3, n=36)*	119.4 (14.3-342.5, 109.7, n=24)	U=284.0, p=0.036
PCT (Procalcitonin (ng/mL))	5.2 (0.1-100, 19.5, n=60)*	1.9 (0.1-76.9, 10.4, n=36)*	9.8 (0.4-100, 36.5, n=24)*	U=247.0, p=0.005
Body temperature (°C)	38.2, (37.0-41.2, 1.1, n=52)*	38.2 (37-39.8, 0.9, n=33)	38.4 (37.5-41.2, 1.2, n=19)*	U=249.0, p=0.219
SOFA (points)	5.4 (2-14.2, 6.5, n=52)*	4.8 (2-12.2, 3.6, n=33)*	10.2 (4.4-14.2, 5.4, n=19)	U=101.0, p<0.001
ΔSOFA (points)	0 (-6 to 9, 4, n=44)	0 (-4 to 3, 3, n=29)	2 (-6 to 9, 4, n=15)	t=-2.5, p=0.018
Creatinine (μmol/L)	105.4 (46-857.2, 116.4, n=60)	88.5 (46-489.8, 51.8, n=36)*	182.5 (61-857.2, 151.5, n=24)*	U=216.5, p=0.001

*non-parametric distribution; the values represent the median (range, IQR, number of patients); Δ-absolute difference between the fifth and the baseline measurements (change from baseline)

Supplementary Table S2. Repeated measures correlations for combined hematological and biochemical variables in study patients.

Correlations[1]	All patients	Survival group (Controls)	Fatality group (Cases)
MDW & CRP	0.318 (209, <0.001*, 0.191-0.435)	0.182 (5, 0.040*, 0.008 to 0.345)	0.476 (83, <0.001*, 0.293-0.626)
MDW & NLR	0.007 (217,0.911, -0.125 to 0.140)	0.091 (131, 0.296, -0.080 to 0.257)	-0.045 (85,0.677, -0.253 to 0.166)
MDW & PCT	0.227 (212, <0.001*, 0.096-0.351)	0.217 (126, 0.014*, 0.045-0.376)	0.244 (85, 0.023*, 0.035 to 0.432)
MDW & PLR	0.189 (217, 0.005*, 0.058-0.314)	0.071, (131, 0.418, -0.101 to 0.238)	0.303 (85, 0.004*, 0.098-0.483)
MDW & SOFA	0.123 (188, 0.090, -0.019 to 0.261)	0.168 (120, 0.063, -0.009 to 0.336)	0.074 (67, 0.545, -0.165 to 0.305)
CRP & NLR	0.182 (212, 0.007*, 0.050-0.309)	0.203 (125, 0.022*, 0.030-0.365)	0.182 (86, 0.089, -0.028 to 0.377)
CRP & PLR	0.298 (212, <0.001*, 0.171-0.416)	0.104 (125, 0.245, -0.071 to 0.273)	0.410 (86, <0.001*, 0.219-0.570)

1- repeated measures correlation, numbers represent the correlation coefficient r (degree of freedom, probability, 95% confidence interval of r)
